# Temperature Responses of Ammonia-Oxidizing Prokaryotes in Freshwater Sediment Microcosms

**DOI:** 10.1371/journal.pone.0100653

**Published:** 2014-06-24

**Authors:** Jin Zeng, Dayong Zhao, Zhongbo Yu, Rui Huang, Qinglong L. Wu

**Affiliations:** 1 State Key Laboratory of Lake Science and Environment, Nanjing Institute of Geography and Limnology, Chinese Academy of Sciences, Nanjing, China; 2 State Key Laboratory of Hydrology-Water Resources and Hydraulic Engineering, Hohai University, Nanjing, China; 3 College of Hydrology and Water Resources, Hohai University, Nanjing, China; Argonne National Laboratory, United States of America

## Abstract

In order to investigate the effects of temperature on the abundances and community compositions of ammonia-oxidizing archaea (AOA) and bacteria (AOB), lake microcosms were constructed and incubated at 15°C, 25°C and 35°C for 40 days, respectively. Temperature exhibited different effects on the abundance and diversity of archaeal and bacterial *amoA* gene. The elevated temperature increased the abundance of archaeal *amoA* gene, whereas the abundance of bacterial *amoA* gene decreased. The highest diversity of bacterial *amoA* gene was found in the 25°C treatment sample. However, the 25°C treatment sample maintained the lowest diversity of archaeal *amoA* gene. Most of the archaeal *amoA* sequences obtained in this study affiliated with the *Nitrosopumilus* cluster. Two sequences obtained from the 15°C treatment samples were affiliated with the *Nitrosotalea* cluster. *N. oligotropha* lineage was the most dominant bacterial *amoA* gene group. Several sequences affiliated to *Nitrosospira* and undefined *N. europaea/NC. mobilis* like lineage were found in the pre-incubation and 25°C treatment groups.

## Introduction

Nitrification coupled with denitrification is of special importance for keeping nutrient balance in the freshwater lake ecosystems by removing nitrogen into the atmosphere [1]–[2]. Nitrification is the biological oxidation of ammonia (NH_3_) into nitrite (NO_2_^−^) followed by the oxidation of nitrite into nitrate (NO_3_^−^). Transformation of ammonia to nitrite is the first and rate-limiting step of nitrification [3]–[4]. For a long time, ammonia-oxidizing bacteria (AOB) were considered as the most important contributors to catalyze nitrite from ammonia under aerobic condition [5]. However, recent studies revealed the existence of ammonia monooxygenase encoding archaea and suggested that they are capable of ammonia oxidation [6]–[7]. These putative ammonia oxidizing archaea (AOA) have recently been proposed to belong to a new phylum, *Thaumarchaeota* [8].

The rate of biological reactions is greatly influenced by temperature. Previous studies indicated that the nitrification rate increases gradually from <10°C to 30°C in the environments [9]–[10]. AOB could grow within a wide temperature range [11]. Fierer et al. [12] investigated 23 different soil samples of the North America and demonstrated that temperature is the most important factor affecting community structure of soil AOB. Horz et al. [13] investigated the effects of global climate change on AOB and found that the abundance of AOB increased with the elevated temperature and the community structures of AOB also changed under different temperatures.

Temperature could also affect the growth, activity, abundance and community structure of AOA. *Nitrososphaera viennensis* exhibits optimal growth at 37°C [14], whereas some thermophilic AOA, for example *Nitrosocaldus yellowstonii*, grow at temperatures up to 74°C [15]. A previous study demonstrated that the abundance and diversity of AOA decreased to the lowest in the aquarium biofiltration system of low temperature (5.5°C) [16]. Tourna et al. [17] reported that the relative abundance and transcriptional activity of ammonia oxidizing bacteria were not significantly changed with temperatures. However, the community structure of active archaeal ammonia oxidizers clearly changed in soil microcosms. The relative abundance of marine and subsurface associated archaea, rather than soil archaea, increased with temperature. Nevertheless, Leininger et al. [18] showed that the quantity of crenarchaeota population is only slightly affected by temperature in the soil ecosystem.

The relationships between environmental factors and the ecological distribution of AOA and AOB have mainly been investigated in soil and marine ecosystems so far [19]–[21]. However, the knowledge gaps about the responses of AOA and AOB to temperature in lake ecosystems need to be filled. In the present study, lake microcosms under different temperatures were constructed in the laboratory. The main objective of this research was to investigate the effects of temperature on the abundances and community compositions of AOA and AOB in lake sediments.

## Materials and Methods

### Construction of Lake Microcosms

Lake Taihu (30°55′40″–31°32′58″N, 119°52′32″–120°36′10″E) is a eutrophic freshwater lake located at the south part of Yangtze Delta in China and has an area of 2338.1 km^2^. The average water depth of the lake is 1.9 m [22]. Lake water and sediment were collected at Meiliang Bay (31°28′46.39˝ N, 120812′05.38˝ E) of Lake Taihu in May of 2012. (This study has been approved by the Taihu Basin Authority of the Chinese Government. The field sampling did not involve endangered, protected species and vertebrate animals.)

Lake microcosms were constructed in cylindrical plexiglass containers (diameter 11.5 cm and height 45 cm), which included the lake water and sediment samples (the water depth was 23 cm and the sediment depth was 20 cm). To simulate the natural lake environment and to facilitate the collection of sediment for subsequent analysis, the top 20 cm of the collected sediment cores were sectioned into 1-cm intervals, and samples at the same depth were pooled together. Sediments were then sieved with a 0.6-mm mesh to remove the macrofauna and large particles. The sieved sediment samples were fully homogenized and placed into plexiglass tubes with 1-cm intervals corresponding to their original depths. Lake water was filtered and added into the plexiglass tubes using intravenous needles. The top 1 cm sediment was collected as the 0 d sample (the pre-incubation sediment). Then, the cylindrical containers were remained under dark environment and stored in the 15°C, 25°C and 35°C (corresponding to the main temperature variation in the Lake Taihu region) incubators for 40 d, respectively. Each temperature group had three replicates. Oxygen concentrations in the overlying water were monitored by a glass electrode (PHB4, REX, China) to ensure the oxic environment during the incubations. After the incubation, the top 1 cm sediment samples were collected and transferred to sterile centrifuge tubes (the three replicates were homogenized). The samples were stored at −70°C until further analysis.

### Analysis of Sediment Properties

The sediment samples were put in the freezing air dryers (ALPHA 1–2, CHRIST, Germany) before analyzing the chemical parameters. The measuring of total nitrogen (TN) and pH were carried out according to Jin and Tu [23]. Other nitrogen associated factors including NH_3_-N and NO_3_-N were extracted by 2 M KCl and measured in the Continuous Flow Analyzer (San++, SKALAR, Netherlands) [24].

### DNA Extraction

DNA was extracted from 250 mg sediment with the PowerSoil DNA Isolation Kit (MoBio Laboratories, Solana Beach, CA) according to the manufacturer's instructions. The extracted DNA was quantified using a spectrophotometer (Eppendorf, Hamburg, Germany) and was diluted with the TE buffer to 5 ng DNA/μl for the quantitative PCR analysis.

### Real-time Quantitative PCR

In order to investigate the effect of temperature on the abundance of AOA and AOB in the surface sediments, real-time PCR was used to analyze the *amoA* gene copy numbers of archaea and bacteria in triplicate in different sediment samples.

The archaeal and bacterial *amoA* genes were amplified using primers Arch-amoAF/Arch-amoAR [25] and amoA-1F/amoA-2R [26], respectively. A series of 10 times diluted plasmids were used to yielded the standard curves. Archaeal *amoA* gene copy numbers were diluted from 3.40×10^8^ to 3.40×10^2^ copies/μl and bacterial *amoA* gene copy numbers were diluted from 1.59×10^8^ to 1.59×10^2^ copies/μl. The PCR amplification was performed on the IQ5 Thermocycler (RG65HD, Corbett, Australia) with the fluorescent dye of SYBR Green I.

The 20 µl reaction mixture, including 5 ng sediment DNA template, 0.2 µM forward and reverse primers, 10 µl 2×SYBR Premix Ex Taq™ buffer (Takara, Japan). The control was always run with water as the template. Thermalcycler protocol for archaeal *amoA* followed the program: 3 min at 95°C; 45 cycles of 30 s at 95°C, 1 min at 53°C, 20 s at 72°C and a final extension of 7 min at 72°C. The PCR amplification program for bacterial *amoA* was 3 min at 95°C; 45 cycles of 30 s at 95°C, 1 min at 55°C, 20 s at 72°C and a final extension of 7 min at 72°C.

Melting curve and 2% agarose gel electrophoresis were employed to check the specificity of PCR products. Data analysis was carried out with the Rotor-Gene 6000 software package. The PCR amplification efficiencies were 0.96–1.03 for archaeal *amoA* and 0.92–0.97 for bacterial *amoA*. The obtained data were calculated with the mass of the sediment samples to determine the number of archaeal and bacterial *amoA* gene copies/g sediment (dry weight).

### Clone Library Construction and Phylogenetic Analysis

In order to investigate the impacts of temperature on the community compositions of AOA and AOB in surface sediments, the *amoA* gene clone libraries were constructed for each sediment sample. PCR amplification of archaeal and bacterial *amoA* genes were performed using the same primers as real-time quantitative PCR. Triplicate 25 µl PCR products were pooled, purified (Axygen PCR cleanup purification kit, Hangzhou, China) and cloned using the pGEM-T vector (Promega, Madison, WI, USA) and competent *Escherichia coli* cells (DH5α, Takara, Japan). Picked transformants were grown overnight on LB agar plates containing 100 µg/ml ampicillin, 40 µg/ml X-Gal and 24 µg/ml IPTG. Positive clones were checked by PCR amplification using vector primers (T7 and SP6). Clones which containing the PCR product inserts were sequenced at the Shanghai Majorbio Bio-technology Co., Ltd.

The obtained DNA sequences were translated into conceptual protein sequences and the online GenBank BLAST program was used for retrieval of the closest matched sequences [27]. Multiple sequence alignments of the most related environmental sequences and clone sequences were carried out by ClustalX [28]. Indices of diversity (Shannon-Weiner, *H*) and nonparametric richness estimator (bias-corrected Chao1 (*S*_Chao1_)) were calculated for each library using the DOTUR software package [29]. The highest quality representative sequence of each OTU was exported for phylogenetic analysis. Neighbor-joining trees were built using the MEGA 4.0 software package [30].

### Nucleotide Sequence Accession Numbers

The sequences obtained in this study were deposited in the GenBank database under accession numbers JX827631-JX827692 (AOA) and JX504547-JX504663 (AOB).

### Statistical Analysis

For multiple comparisons, one-way ANOVA with Tukey's post hoc tests were performed for multiple comparisons with the SPSS 13.0 package (SPSS). *P* values of 0.05 were selected for significance.

## Results

### Sediment Chemical Properties

As shown in Fig. S1, pH of the 15°C treatment sediment sample (pH = 5.78) was significantly lower than the other samples. The amount of TN and NH_3_-N correlated negatively with temperature. Compared with the pre-incubation samples, slight reductions in TN concentrations were observed in all the treatment groups after 40 d. The 35°C treatment samples maintained the lowest TN concentration of 0.78 mg/g. The concentrations of NH_3_-N also decreased after incubation, indicating the consumption of ammonia in sediment samples. Significant accumulations of NO_3_-N were observed for all treatment groups, suggesting the strong nitrification activities.

### Real-time Quantitative PCR

**Figure 1:**
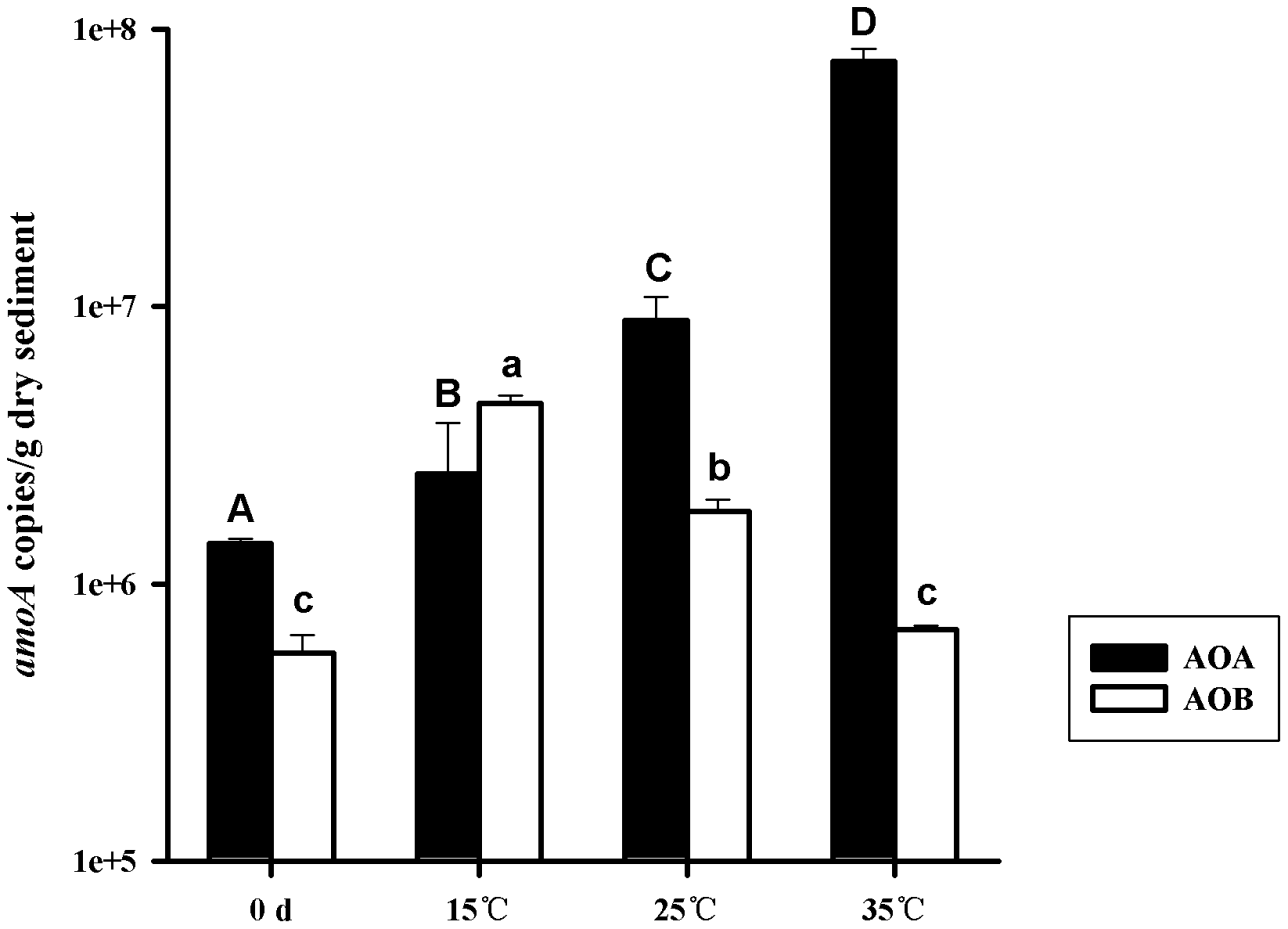
Abundances of archaeal and bacterial *amoA* genes in the surface sediment samples treated with different temperatures. Data are shown as Means±SD (n = 3). Different uppercase (archaeal *amoA* gene) and lowercase letters (bacterial *amoA* gene) refer to significant differences between different temperature treatments (*P*<0.05).

The abundances of AOA and AOB in the sediment samples were characterized by using the real-time quantitative PCR analysis of the archaeal and bacterial *amoA* gene copy numbers. The abundances of archaeal *amoA* gene were significantly higher than those of bacterial *amoA* gene for all samples except for the 15°C treatment (Fig. 1). The lowest abundance of archaeal *amoA* gene (1.40×10^6^ copies/g dry sediment) was detected in the 0 d sediment sample. Then, the archaeal *amoA* gene abundances increased along with the temperature after 40 d incubation (increased from 2.50×10^6^ to 7.63×10^7^ copies/g dry sediment).

The abundance of bacterial *amoA* gene exhibited negative correlation with temperature (Fig. 1). The highest abundance of bacterial *amoA* gene (4.48×10^6^ copies/g dry sediment) was detected in the 15°C treatment samples.

### Diversity of AOA and AOB

Four clone libraries, including 62 archaeal *amoA* DNA sequences, were analyzed. Each library included 10–25 sequences (Table 1). OTUs were identified based on the 5% cut-off at the DNA sequence level. Shannon-Weiner (*H*) and S_Chao1_ indexes were calculated and shown in Table 1. Diversity of the archaeal *amoA* gene in the 15°C treatment samples was the highest, followed by the 35°C treatment samples. The AOA diversities of 0 d and 25°C treatment samples were the lowest, both of which contained only 1 OTU.

**Table 1:** Diversity indices of archaeal and bacterial *amoA* clone libraries derived from sediment samples incubated at different temperatures^a^.

Clone libraries	NO. of clones	NO. of OTUs	*H*	S_Chao1_
**AOA**				
0 d	10	1	0.000	1
15°C	13	2	0.429	2
25°C	14	1	0.000	1
35°C	25	2	0.168	2
Total	62	3	0.225	3
**AOB**				
0 d	29	4	0.977	4
15°C	30	2	0.500	2
25°C	28	5	0.997	5.5
35°C	30	4	0.674	5
Total	117	6	0.861	6

a OTUs were defined as 5% difference in nucleic acid sequences. Shannon-Weiner (*H*)and S_Chao1_-estimated richness was calculated using DOTUR.

For bacterial *amoA* gene, four clone libraries were generated and a total of 117 clones were sequenced, which could be divided into 6 OTUs. Each library contained 2–5 OTUs. The 25°C treatment samples maintained the highest Shannon-Weiner (*H*) indexes and the number of OTUs. The 0 d and 35°C treatment samples maintained medium diversity of AOB. Only two OTUs were observed in the clone library of 15°C treatment samples (Table 1).

### Phylogenetic Analysis of Archaeal and Bacterial *amoA* genes

**Figure 2:**
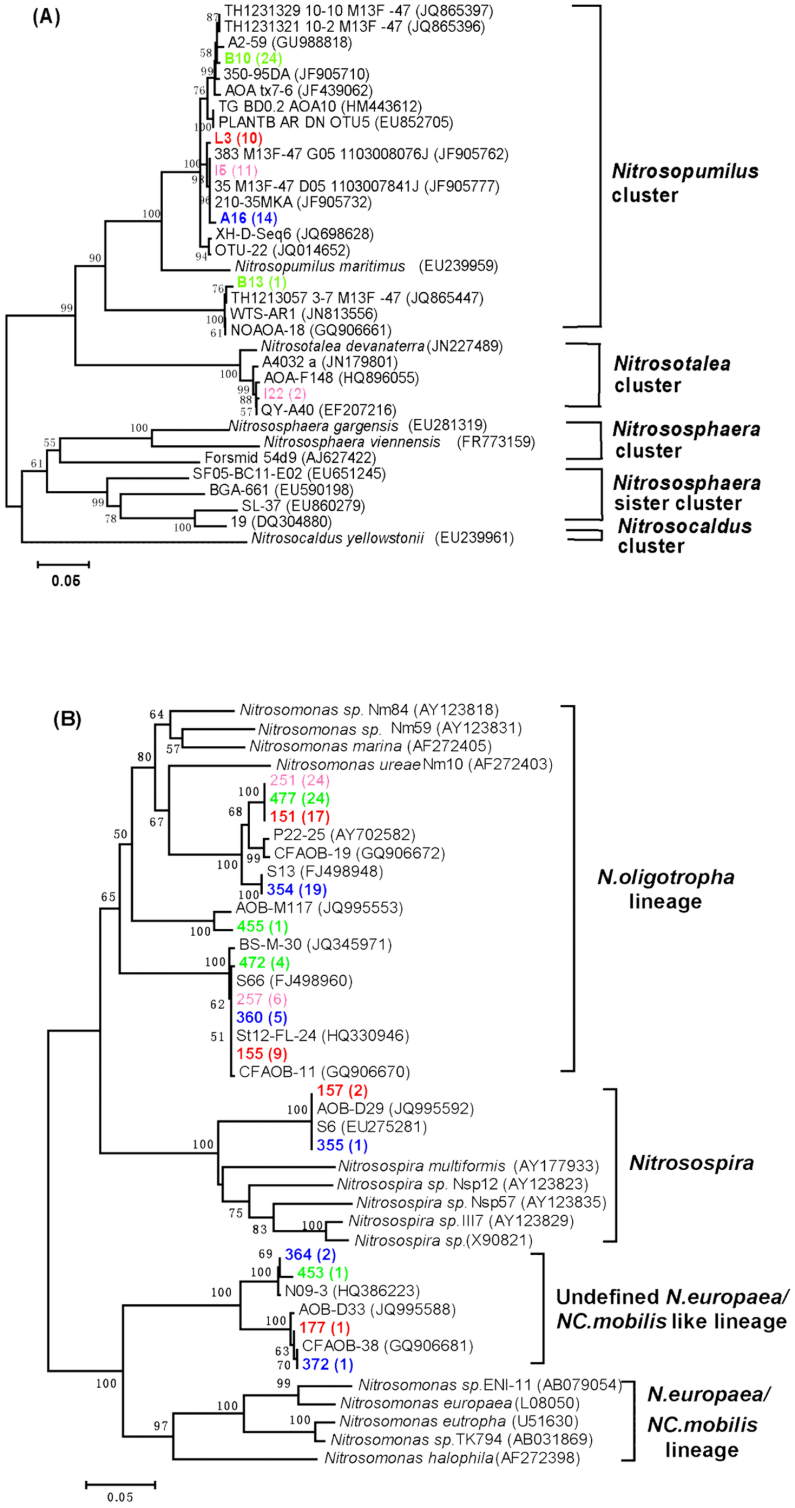
Phylogenetic trees of the archaeal (A) and bacterial (B) *amoA* gene sequences. Sequences obtained in this study were written in bold and colors, which represent different clone libraries (Red: 0 d; pink: 15°C treatment; blue: 25°C treatment; green: 35°C treatment). One representative sequence from each OTU is shown in the phylogenetic trees. Numbers in parentheses indicate the number of sequences affiliated to the same OTU. Bootstrap numbers >50% are shown.

Figure 2A shows the phylogenetic tree of the archaeal *amoA* gene sequences obtained from different samples of this study. Pester et al. [31] reported that archaeal *amoA* sequences fell into five clusters, *Nitrosopumilus* cluster, *Nitrosotalea* cluster, *Nitrosocaldus* cluster, *Nitrososphaera* cluster and *Nitrososphaera* sister cluster. The archaeal *amoA* sequences recovered in this study were mainly affiliated within the *Nitrosopumilus* cluster (Fig. 2A).

**Figure 3:**
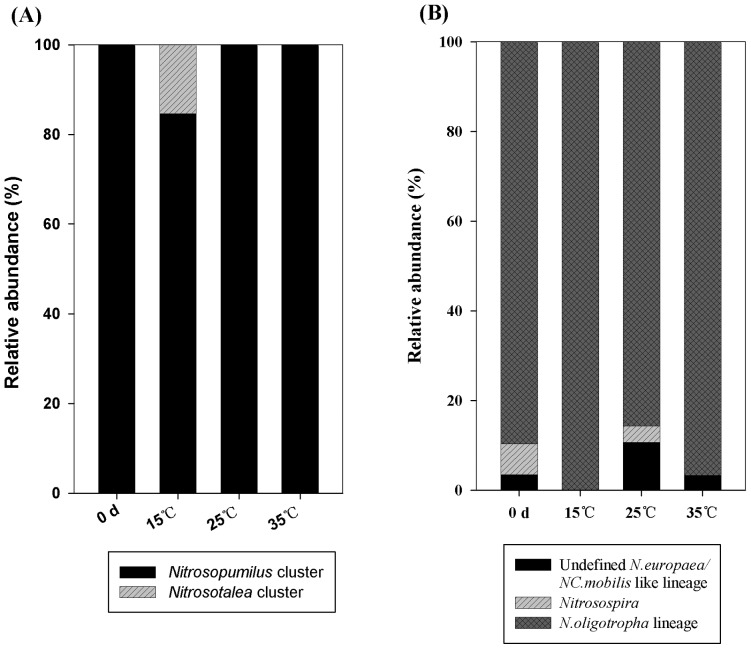
Relative abundances of the different groups of archaeal (A) and bacterial (B) *amoA* genes in sediment samples treated with different temperatures.

All the archaeal *amoA* gene sequences derived from 0 d, 25°C and 35°C treatment samples were affiliated with *Nitrosopumilus* cluster, which covered 96.77% of the total number of the archaeal *amoA* sequences obtained in this study. Most of the archaeal *amoA* sequences recovered from clone libraries of 15°C (84.62%) treatment samples were also affiliated with *Nitrosopumilus* cluster (Fig. 3A). Only two sequences obtained from clone libraries of 15°C treatment samples were affiliated with the *Nitrosotalea* cluster.

Sequences affiliated with *Nitrosopumilus* cluster were mainly closely related to the sequences derived from sediments of eutrophic lake (JF905732 and JF905777), the Three Gorges Dam of the Yangtze River (HM443612) and the sewage treatment plant (JQ865396). One OTU recovered from the 35°C treatment samples was affiliated with the AOA sequences isolated from estuarine sediment of the Mississippi River (GQ906661) and the sewage treatment plant (JQ865447).

The bacterial *amoA* sequences recovered in this study mainly fell into two clusters, *N. oligotropha* lineage and *Nitrosospira*, which were commonly found AOB in the freshwater ecosystems [11] (Fig. 2B). In addition, five sequences belonged to an undefined *N. europaea/NC. mobilis* like lineage lacking cultivated representatives. Differences in the community compositions of bacterial ammonia oxidizers in different treatment groups were observed. The microcosms incubated at 15°C were exclusively composed of AOB closely related to the *N. oligotropha* lineage. 6.90% and 3.57% of the sequences retrieved from 0 d and 25°C treatment group fell within the *Nitrosospira* lineage, while no sequences was found within this lineage in the other two groups.

*N. oligotropha* lineage was the dominated group among the three clusters, which covered 93.16% of the total AOB sequences obtained in this study. All of the bacterial *amoA* sequences derived from the 15°C treatment samples and 96.67% of the bacterial *amoA* sequences recovered from the 35°C treatment samples were affiliated with the *N. oligotropha* lineage (Fig. 3B). In the phylogenetic tree, this lineage had higher similarities with sequences isolated from the estuarine sediments of the Mississippi River (GQ906672 and GQ906670), sediment of a small eutrophic urban lake, Moon Lake of Hubei province (FJ498948 and FJ498960) and Shanghai Dongtan wetlands (JQ345971). Cluster *Nitrosospira* included two sequences from the 0 d clone library and one sequence from the 25°C treatment clone library. This cluster affiliated with sequence derived from the sediments of large shallow eutrophic Lake Taihu (JQ995592). Only 4.27% of the total AOB sequences affiliated with the Undefined *N. europaea/NC. mobilis* like lineage and these sequences had higher similarities with the sequences isolated from the Mississippi River (GQ906681) and the sediments of eastern Lake Taihu (JQ995588).

## Discussion

### Effects of Temperature on the Abundance and Community Compositions of AOA and AOB

In the present study, the abundances of archaeal *amoA* gene showed positive correlations with the temperature. Cao et al. [32] investigated the abundances of archaeal and bacterial *amoA* genes in surface sediments from the coastal Pearl River estuary to the South China Sea (The temperature of the sediment samples ranged from 2.9°C to 21.3°C). No significant correlation was found between the temperature and the abundances of both archaeal and bacterial *amoA* genes. Wu et al. [33] found the abundances of archaeal *amoA* gene were comparable in the 15°C and 25°C treatment groups, however, a significant decline in the abundance of archaeal *amoA* gene was found in the 37°C treatment group after 4-week incubation. The diversity of the archaeal *amoA* gene in this study was not significantly correlated with the temperature, which was consistent with previous study [32]. Zhao et al. [34] found the Chao1 richness indexes of the archaeal *amoA* gene in sediments of three hot springs (42°C to 87°C) were more frequently higher at temperatures below 75°C than above it, indicating that AOA may be favored in the moderately high temperature environments. In the present study, one sequence of the archaeal *amoA* gene derived from the 15°C treatment sample was affiliated with sequences recovered from the red soils (HQ896055 and EF207216). This sequence was affiliated with the *Nitrosotalea* cluster, which contained an autotrophic, obligately acidophilic ammonia oxidizing thaumarchaeon, *Nitrosotalea devanaterra*, isolated from acid soils [35]. This was consistent with the lower pH values found in the 15°C treatment sample.

According to the results of the real-time PCR, significant growth of AOB could be found in the 15°C and 25°C treatment samples. Previous study has demonstrated that AOB could grow at low temperatures [11]. The results were consistent with previous reports that bacterial *amoA* gene abundance was negatively related with the temperature (r = −0.779, *P*<0.001) after incubation for 4 weeks [33]. Elevated temperature would enhance the decomposition of extracellular DNA or dead cells in sediment [33], which may explain the lower AOB abundance in the 35°C treatment samples. In the present study, the diversity of AOB was the highest in the 25°C treatment group. Cao et al. [32] found a negative correlation between the temperature (from 2.9°C to 21.3°C) and the diversity of the bacterial *amoA* gene in surface sediments from the coastal Pearl River estuary to the South China Sea. The AOB sequences obtained in the present study were mostly belonged to *N. oligotropha* lineage, which is often found in freshwater lakes, but its ecophysiological traits remain unclear. Additionally, several sequences affiliated to *Nitrosospira* and undefined *N. europaea/NC. mobilis* like lineage were found in the 0 d and 25°C treatment groups. The pre-incubation samples (0 d) were collected in May of 2012 and the water temperature at the sampling station was around 25°C.

Previous studies have demonstrated the significant differences of the effects of temperature on the ammonia oxidizing archaea and bacteria. The moderate thermophile AOA, *Candidatus Nitrososphaera gargensis*, enriched from a Siberian hot spring, has an optimum growth temperature of about 46°C [36]. Some species of AOA isolated from terrestrial hot springs (*Nitrosocaldus yellowstonii*) can grow at temperatures as high as 74°C [15]. Continued surveys of geothermal habitats demonstrated that some species of AOA distributed in hot springs with temperatures as high as 97°C [37]. However, AOB have so far not been detected in environments experiencing constant temperatures of >40°C [38].

### Indirect Factors Influencing the AOA and AOB

In addition to the direct influences, variations in temperature could lead to the fluctuations of other environmental factors, such as, organic carbon, oxygen availability and ammonia, which might also contribute to the community shifts of ammonia oxidizers. Elevated temperature could increase the decomposition rate of organic matter and release more organic molecule, which could also affect AOB. Racz et al. [39] reported that organic carbon such as, peptone and glucose could affect the abundance and diversity of AOB in a mixed culture with the heterotroph community. Previous study has demonstrated the effect of dissolved oxygen on ammonia-oxidizing bacterial communities [40]. The elevated temperature would increase the decomposition rates of organic matter and decrease the O_2_ availability due to heterotrophic bacteria, which have higher affinities for O_2_ than the nitrifiers [41]. The elevated decomposition rate of organic matter would also increase the ammonia availability, which is the substrate for nitrification [42].

### Ecological Significances of Variations in the Communities of AOA and AOB

Variation in temperature would also possibly affect the functions of the ammonia oxidizing prokaryotes. Tourna et al. [17] reported that the community structure of the active archaeal ammonia oxidizers clearly changed in the soil microcosms incubated under different temperatures (10°C–30°C). The relative abundance and transcriptional activity of the marine and subsurface associated archaea (mostly group I.1a related) increased with temperature. However, the relative abundance and transcriptional activity of AOB were not significantly changed with temperatures. Offre et al. [43] also found the changes in the relative abundance of the specific archaeal *amoA* genes during active nitrification in soil microcosms incubated at 30°C. In the present study, the copy numbers of the archaeal *amoA* gene increased significantly in the microcosms incubated at 35°C. Due to the lack of the relative contribution analysis of archaeal and bacterial ammonia oxidizers, we do not yet know whether this increase in the abundance of archaeal *amoA* gene would affect the nitrification rates in the sediment samples. Further studies are needed to investigate the effects of ammonia oxidation inhibitor (acetylene) on the growth of ammonia oxidizer and nitrification kinetics and elucidate the relative contributions of archaeal and bacterial ammonia oxidizers in the freshwater ecosystem under different temperature conditions.

The coupled nitrification-denitrification processes could remove high percentages of anthropogenic nitrogen pollution [44], which is of particular significance in the eutrophic lake ecosystem. With the increasing temperature, ammonia oxidation rate and the nitrite production rate increased, which could result in the NH_3_-N consumption and NO_3_-N accumulation. In the present study, the concentrations of NH_3_-N reduced after incubation, indicating the consumption of ammonia through nitrification in sediment samples. Stark [9] investigated the influence of temperature on ammonia oxidation and reported that the optimum temperature for potential nitrification was in the range of 20°C–37°C. Avrahami et al. [45] also reported a decrease in ammonium concentration in microcosms of an agricultural soil after short (5 days) and long (6.5, 16 and 20 weeks) incubation. In the present study, the amount of TN decreased in all treatment groups compared to the pre-incubation samples suggesting the nitrogen loss after incubation. The remained TN amount correlated negatively with temperature. Higher temperatures would induce the decreased dissolved oxygen in the sediments, which could activate anaerobes (such as denitrifiers) and the denitrification activity, promoting the NO_3_-N consumption and N_2_O production. Avrahami et al. [45] also demonstrated the release rates of N_2_O increased monotonously between 4 and 37°C in the soil microcosms. This was also confirmed by the accumulation of NO_3_-N in the 35°C treatment that was not significantly higher than the other two groups in the present study.

Variation in the temperature could change the abundances and community compositions of AOA and AOB in the lake sediments and therefore affect the nitrogen cycling process. The coupled nitrification-denitrification processes play pivotal roles in removing the nitrogen from the eutrophic lake ecosystem. At present, there have been several studies reported the variations in the community compositions of archaeal and bacterial ammonia oxidizer along the eutrophic gradient [4], [46]–[47]. Hou et al. [4] also indicated that AOA may play a less important role than AOB in the nitrification process of eutrophic lakes. Future studies are needed to investigate the effects of temperature induced variation in the community compositions of ammonia oxidizer on the lake eutrophication.

## Conclusions

In summary, the effects of temperature on the abundance and community composition of AOA and AOB in the eutrophic lake microcosms were investigated. Different effects of temperature on the abundance and diversity of archaeal and bacterial *amoA* gene were observed. The elevated temperature increased the abundance of archaeal *amoA* gene, whereas the abundance of bacterial *amoA* gene decreased. The diversity of AOB was higher than that of AOA. The highest diversity of AOB was found in the 25°C treatment sample. However, the 25°C treatment sample maintained the lowest diversity of AOA. Most of the archaeal *amoA* sequences obtained in this study affiliated with the *Nitrosopumilus* cluster. *N. oligotropha* lineage was the dominant bacterial *amoA* group. The information obtained in this study would be useful to elucidate the temperature response mechanisms of nitrogen cycle in the eutrophic lake ecosystem.

## Supporting Information

Figure S1 **Physicochemical properties of the surface sediment samples incubated at different temperatures.** (A) pH; (B) total nitrogen, TN; (C) NH_3_-N; (D) NO_3_-N. Data are shown as Means ± SD (n = 3). Different superscript letters refer to significant differences between the samples (*P*<0.05).
(TIF)
