# Supplementary figures and images for: Temperature Responses of Ammonia-Oxidizing Prokaryotes in Freshwater Sediment Microcosms
Source: PLoS One. 2014 Jun 24;9(6):e100653. doi: 10.1371/journal.pone.0100653 (PMC4069112; doi:10.1371/journal.pone.0100653)

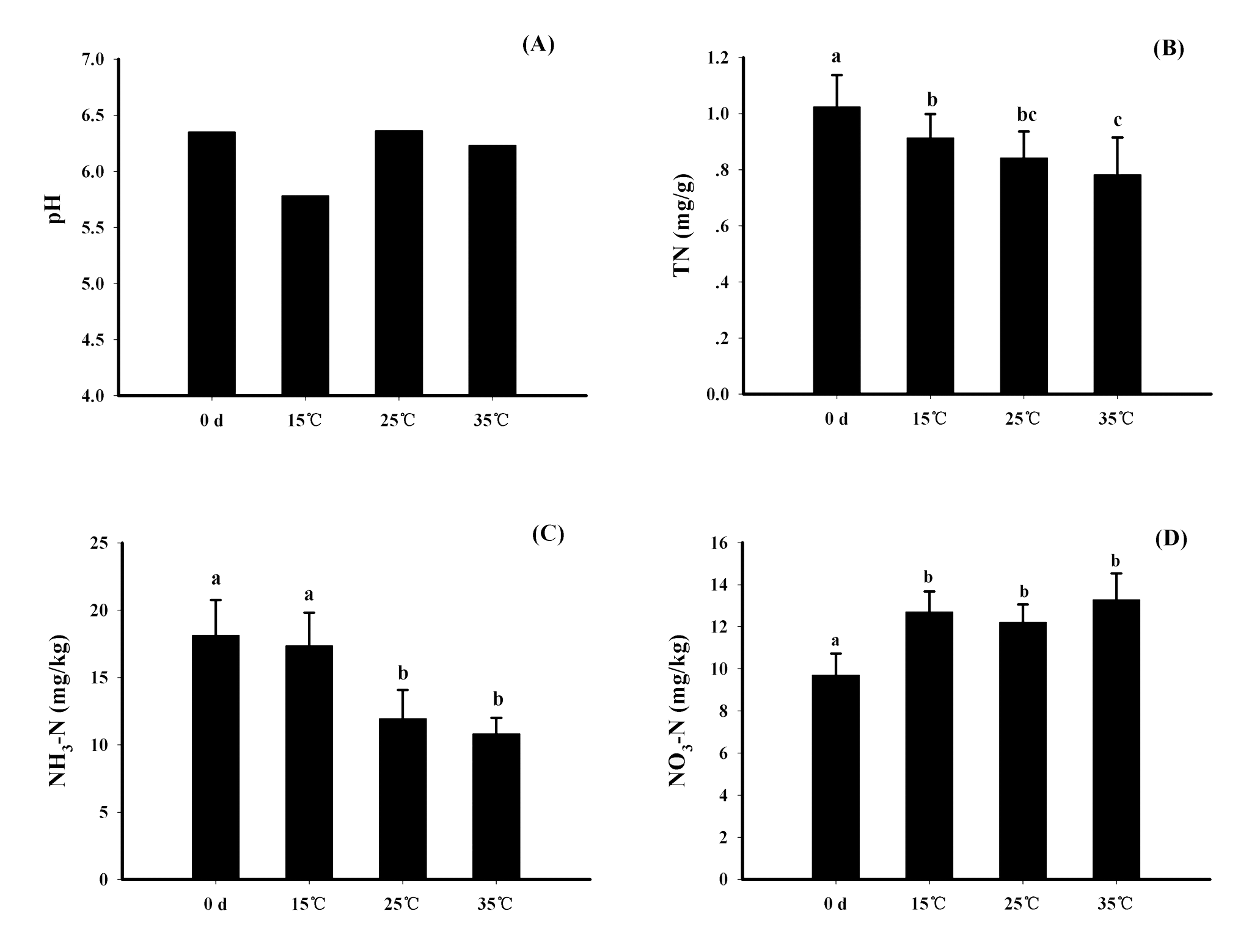

Supplement: Figure S1 — Physicochemical properties of the surface sediment samples incubated at different temperatures. (A) pH; (B) total nitrogen, TN; (C) NH3-N; (D) NO3-N. Data are shown as Means ± SD (n = 3). Different superscript letters refer to significant differences between the samples (P<0.05). (TIF) [file pone.0100653.s001.tif]
